# Impact of DNA double-strand breaks on pancreaticobiliary maljunction carcinogenesis

**DOI:** 10.1186/s13000-021-01132-0

**Published:** 2021-08-09

**Authors:** Yasuhiro Kuraishi, Takeshi Uehara, Takashi Muraki, Mai Iwaya, Yasuhiro Kinugawa, Tomoyuki Nakajima, Takayuki Watanabe, Yusuke Miyagawa, Takeji Umemura

**Affiliations:** 1 Department of Gastroenterology, Shinshu University School of Medicine, Matsumoto, Japan; 2 Department of Laboratory Medicine, Shinshu University School of Medicine, Matsumoto, Japan; 3 Department of Gastroenterology, North Alps Medical Center Azumi Hospital, Ikeda, Japan; 4 Department of Surgery, Shinshu University School of Medicine, Matsumoto, Japan; 5 Department of Life Innovation, Institute for Biomedical Sciences, Shinshu University School of Medicine, Matsumoto, Japan

**Keywords:** Carcinogenesis, DNA double-strand break, Gallbladder, Pancreaticobiliary maljunction, γ-H2AX

## Abstract

**Background:**

Pancreaticobiliary maljunction (PBM) is a condition characterized by chronic inflammation due to refluxed pancreatic juice into the biliary tract that is associated with an elevated risk of biliary tract cancer. DNA double-strand breaks (DSBs) are considered the most serious form of DNA damage. DSBs are provoked by inflammatory cell damage and are recognized as an important oncogenic event in several cancers. This study used γ-H2AX, an established marker of DSB formation, to evaluate the impact of DNA damage on carcinogenesis in PBM.

**Methods:**

We investigated γ-H2AX expression immunohistochemically in gallbladder epithelium samples obtained from 71 PBM cases and 19 control cases.

**Results:**

Fourteen PBM cases with gallbladder adenocarcinoma were evaluated at non-neoplastic regions. A wide range of nuclear γ-H2AX staining was detected in all PBM and control specimens. γ-H2AX expression was significantly higher in PBM cases versus controls (median γ-H2AX-positive proportion: 14.4 % vs. 4.4 %, *p* = 0.001). Among the PBM cases, γ-H2AX expression was significantly higher in patients with carcinoma than in those without (median γ-H2AX-positive proportion: 21.4 % vs. 11.0 %, *p* = 0.031).

**Conclusions:**

DSBs occurred significantly more abundantly in the PBM gallbladder mucosa, especially in the context of cancer, indicating an involvement in PBM-related carcinogenesis.

## Background

Pancreaticobiliary maljunction (PBM) is a congenital malformation that is widely recognized as an important risk factor for biliary tract cancer [1, 2]. Under normal circumstances, the pancreatic and common bile ducts join anatomically inside the duodenal wall, with the sphincter of Oddi regulating the outflow of bile and pancreatic juice. In PBM, however, the junction of the pancreatic and common bile ducts is located outside the duodenal wall, where the sphincter of Oddi cannot function. Because the intraductal pressure of the pancreatic duct is normally higher than that of the bile duct, in PBM pancreatic juice frequently refluxes into the biliary duct and gallbladder. In fact, increased concentrations of amylase have been reported in bile obtained from the gallbladder in PBM patients [3, 4]. The refluxed proteolytic pancreatic juice becomes activated in the biliary duct to generate strongly cytotoxic substances, which subsequently induce persistent injury of the biliary tract epithelium. The chronic inflammation caused by the cycle of damage and repair of the biliary tract evokes such histological changes as hyperplasia, metaplasia, and dysplasia, ultimately resulting in an elevated prevalence of biliary tract cancer.

PBM is divided into two major types on the basis of whether the extrahepatic bile ducts are dilated: PBM with biliary dilation (congenital biliary dilation characterized by local dilation of the extrahepatic bile duct) and PBM without biliary dilation [1]. PBM patients with biliary dilation have a high prevalence of bile duct and gallbladder cancers, whereas those without dilation exhibit a high tendency toward gallbladder cancer. Once PBM is identified, prophylactic surgery such as cholecystectomy or excision of the extrahepatic biliary tract with biliary reconstruction is recommended to decrease the risk of cancer.

DNA double-strand breaks (DSBs) and failure of the DNA damage response (DDR) are important events contributing to carcinogenesis in human cells [5–8]. As the most serious form of DNA damage inducing instability of the human genome, DSBs are provoked by cell damage from ionizing radiation, ultraviolet rays, and specific chemical agents [6]. The reactive oxygen species produced by activated neutrophils and macrophages during inflammation contribute to DSBs as well, thus implicating inflammatory events in the DSBs process [9, 10]. Recurrent and persistent chronic inflammation is widely recognized as a prominent causative factor in the oncogenesis of many types of cancers [11, 12]. DSBs from inflammation have been reported to promote human cancer initiation and progression by inducing genomic instability [13]. DSBs and subsequent DDR have also been shown to be activated during the early stages of carcinogenesis in human solid tumors [5].

Ser139 phosphorylation of histone H2AX (γ-H2AX), a minor component of histone H2A, has recently been proposed as an early event after the occurrence of a DSB [14]. Although the exact function of γ-H2AX remains unclear, it reportedly enables DDR by contributing to the recruitment and localization of DNA repair proteins [15]. γ-H2AX represents discrete foci in nuclei that stain immunohistochemically. The immunohistochemical detection of a single γ-H2AX focus is highly sensitive and reliable, with each focus presumed to represent a single DSB. The number of γ-H2AX foci has been correlated with the amount of DSBs induced by ionizing radiation [16].

The carcinogenetic process in PBM has been associated with chronic inflammation due to the persistent reflux of pancreatic juice into the biliary duct and ensuing DNA damage, although the precise mechanism of tumor onset has not been fully elucidated. To the best of our knowledge, there are no reports directly evaluating the relationship between DNA damage, especially in the form of DSBs, and carcinogenesis in PBM. This study aimed to clarify the association between the degree of DNA damage in PBM and gallbladder cancer carcinogenesis using γ-H2AX, an excellent marker of DSB formation.

## Methods

### Case selection

The present study adhered to the current ethical guidelines of the Declaration of Helsinki and was conducted in accordance with the requirements of the Institutional Review Board of Shinshu University School of Medicine (approval number: 537).

Gallbladder tissues resected for the diagnosis of PBM were subjected to immunohistochemical examinations. Between 2000 and 2018, 71 patients with PBM underwent surgical gallbladder resection at Shinshu University School of Medicine or affiliated institutions. Of these, 14 had accompanying gallbladder adenocarcinoma. Nineteen patients who received cholecystectomy between 2016 and 2018 at our institution were adopted as the control group; all had adequate radiological and pathological records confirming the absence of PBM and gallbladder cancer. As the PBM cases with gallbladder adenocarcinoma were investigated only at non-neoplastic portions surrounding neoplastic areas, all PBM gallbladder tissue samples were considered as evaluating noncancerous mucosa. The PBM cases were also divided into two groups on the basis of the presence (14 cases) or absence (57 cases) of gallbladder adenocarcinoma. The diagnosis of PBM was made in accordance with the Japanese diagnostic criteria for PBM 2013 [17], for which an abnormally long common channel and an abnormal union between the pancreatic and bile ducts were confirmed using endoscopic retrograde cholangiopancreatography or magnetic resonance cholangiopancreatography. Sex, age, and the presence of biliary dilation were assessed as factors potentially associated with DNA damage in the gallbladder epithelium of PBM. For biliary dilation, PBM patients were classified as either having dilation (≥ 1.0 cm) or non-dilation (< 1.0 cm).

### Immunohistochemistry and histologic analysis

Immunohistochemistry for γ-H2AX (phospho Ser139, clone EP854[2]Y, Abcam, Cambridge, UK) and p53 (clone DO-7, DAKO, Copenhagen, Denmark) was performed using the EnVision method (DAKO, Hamburg, Germany). Before immunostaining, sections were incubated with microwave irradiation in 0.45 % Tris 5 mM EDTA for 25 min to retrieve antigens, and endogenous peroxidase activity was blocked by 3 % hydrogen peroxide in methanol.

The degree of immunostaining was evaluated and scored by two pathologists. Brown staining in the nucleus of the epithelial cells indicated positive staining of γ-H2AX. We compared the percentages of positive cells, as reported by Sato et al. [18]. The number of cells with a positive-stained nucleus and total cells were counted in three representative high-power fields for each case. Positive-stained cells were then expressed as the percentage of total cells for each field and averaged for the three fields.

To evaluate the relationship between carcinogenesis and DSB formation, we compared the degree of γ-H2AX expression between cases with PBM and the control group, and between PBM cases with and without gallbladder cancer. Similar evaluations were performed on the immunohistochemical results for p53 protein expression. In PBM cases, we also investigated the association between the degree of γ-H2AX expression and such clinical parameters as sex, age, and the presence of biliary dilation.

### Statistical analyses

Mann–Whitney U tests were adopted to test for differences between subgroups of cases. A p-value of < 0.05 was considered statistically significant. All statistical analyses were performed using JMP Statistics software version 13 (JMP, Tokyo, Japan).

## Results

### Clinicopathologic findings

The median age of the PBM patients at the time of gallbladder resection was 52 years (range: 14–81 years). The group contained 19 males (27 %) and 52 females (73 %). The median age of the 13 male (68 %) and six female (32 %) subjects in the control group was 64 years (range: 16–84 years). Among the PBM patients, three individuals developed bile duct carcinoma, while none of them had synchronous gallbladder carcinoma.

### Strong staining of γ-H2AX was observed more frequently in PBM cases

**Fig. 1:**
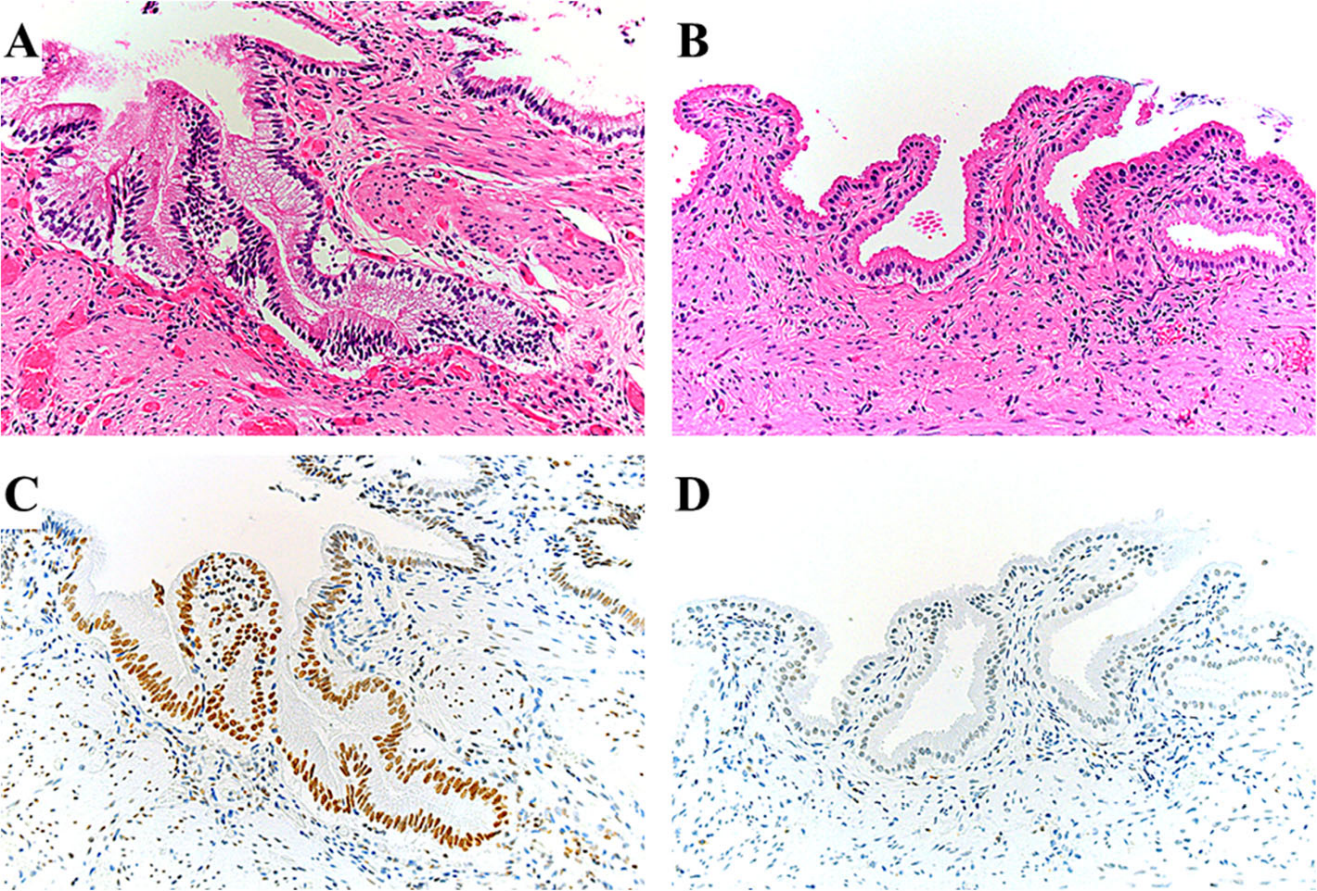
γH2AX expression in the gallbladder tissue of patients with and without pancreaticobiliary maljunction (PBM). Representative hematoxylin-eosin-stained images of (**A**) PBM tissue and (**B**) control tissue. Immunohistochemical staining of γ-H2AX in (**C**) PBM tissue and (**D**) control tissue. Strong staining of γ-H2AX was observed more frequently in the gallbladder epithelium of PBM cases, whereas weak staining was predominantly found in control cases. γ-H2AX, phosphorylated H2AX at Ser139 (200x magnification)

**Fig. 2:**
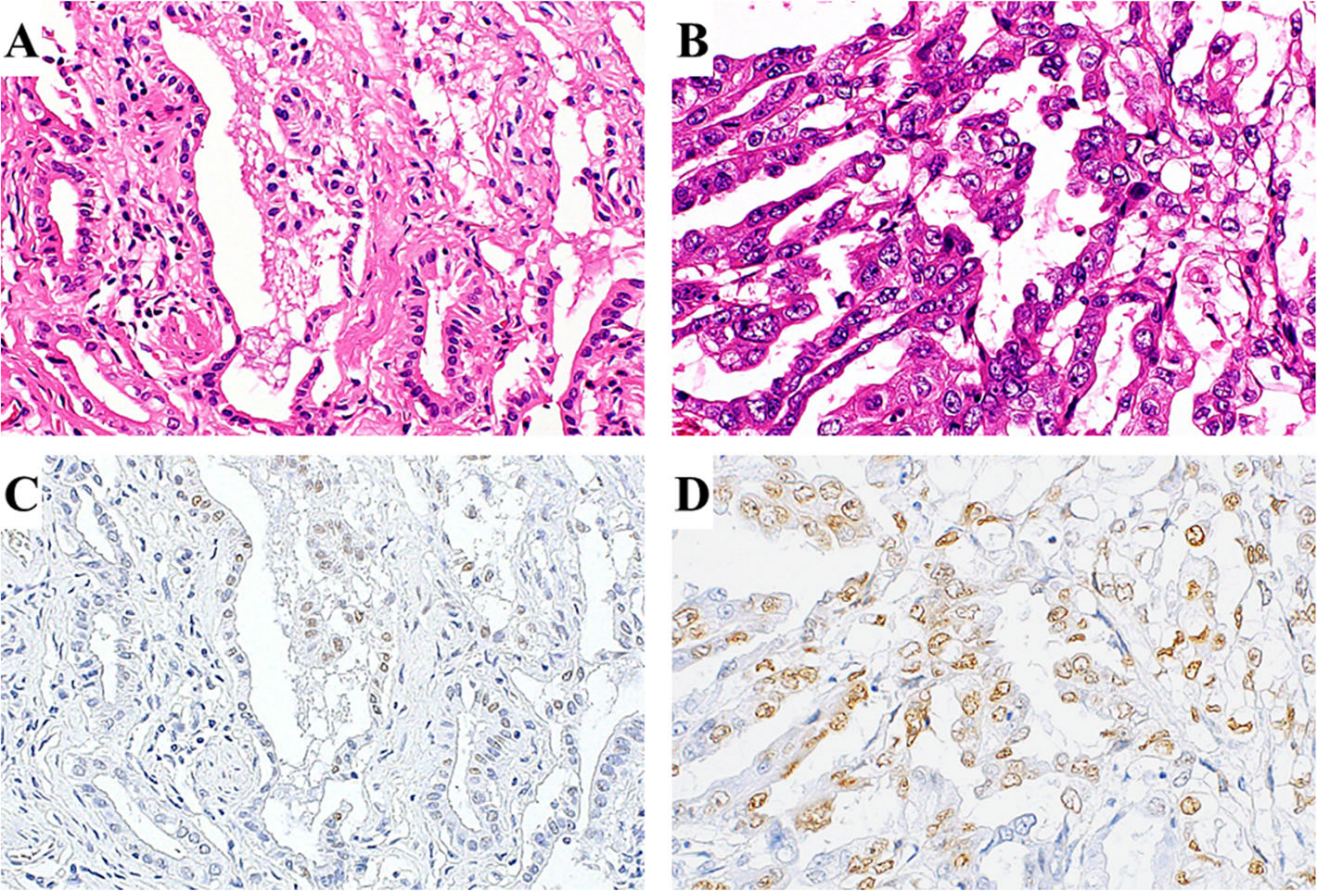
γ-H2AX expression in cancerous and non-cancerous areas of gallbladder tissue in patients with pancreatobiliary maljunction (PBM). Representative hematoxylin and eosin-stained images of the (**A**) non-cancerous area and (**B**) cancerous area. Immunohistochemical staining of γ-H2AX in the (**C**) non-cancerous area and (**D**) cancerous area. The cancerous area appeared to have stronger and more diffuse staining than the adjacent non-cancerous area. γ-H2AX, phosphorylated H2AX at Ser139 (200x magnification)

Cells with a γ-H2AX positive-stained nucleus were present in all PBM and control samples, with a wide range of γ-H2AX-postive cell staining. Figure 1 presents representative histological images of the PBM and control gallbladder epithelium. Strong staining of γ-H2AX was observed more frequently in the gallbladder epithelium of PBM cases, whereas weak staining was more predominant in control cases. Figure 2 shows representative histological images of the adjacent non-cancerous and cancerous tissues in the gallbladder of the same PBM case. The cancerous tissue appeared to have stronger and more diffuse staining than the adjacent non-cancerous tissue.

### γ-H2AX expression was stronger in PBM cases with carcinoma

**Fig. 3:**
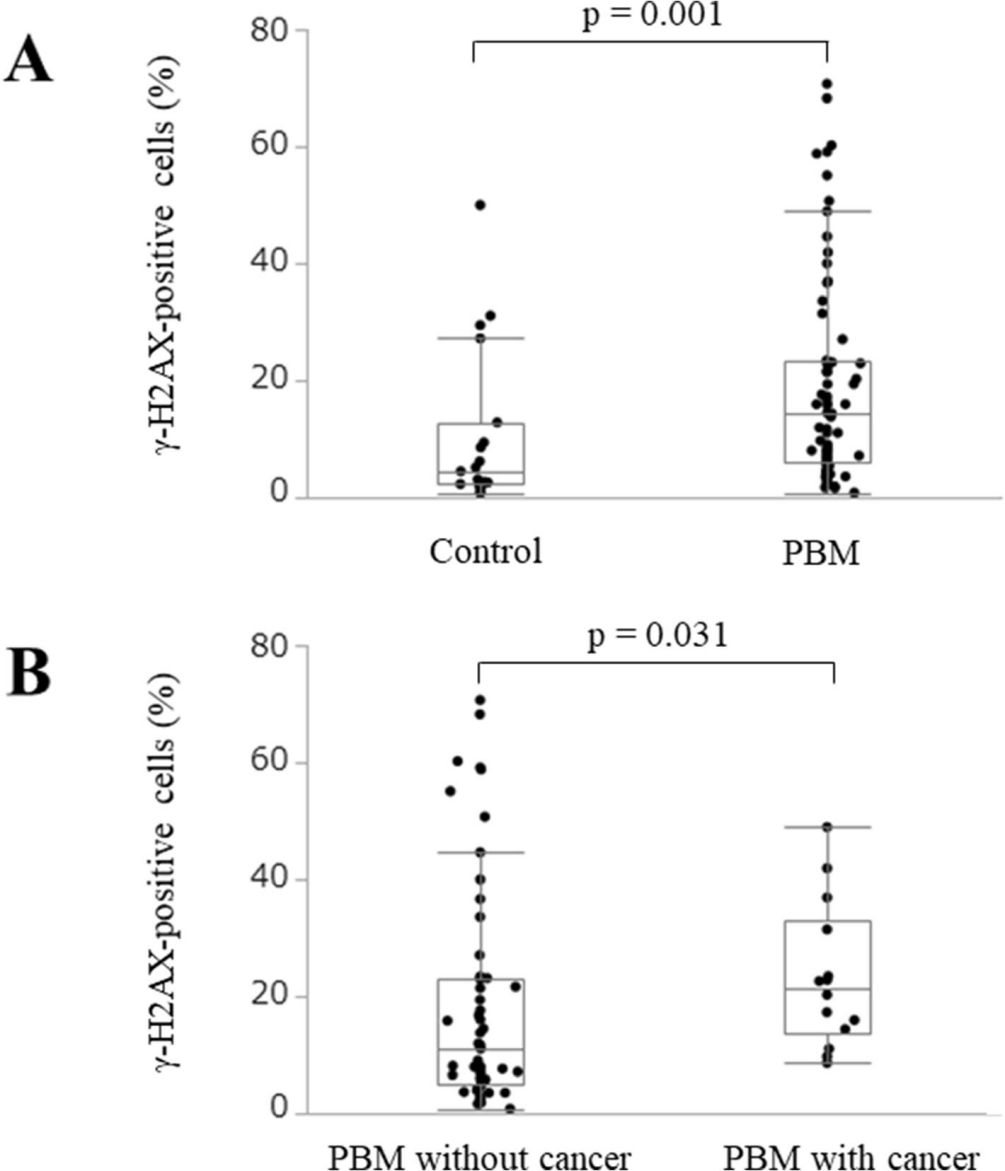
Comparisons of the positive proportion of γ-H2AX expression. **A** The expression of γ-H2AX was significantly higher in cases with PBM than in the control group (*p* = 0.001). **B** The expression of γ-H2AX was significantly higher in PBM cases with carcinoma than in those without (*p* = 0.031). γ-H2AX, phosphorylated H2AX at Ser139; PBM, pancreaticobiliary maljunction

In PBM epithelial cells, a considerable amount of DSBs were visible, presumably due to the chronic inflammatory damage caused by refluxed pancreatic juice in the biliary duct. To evaluate the degree of DSB formation in the PBM gallbladder, we compared the positive proportions of γ-H2AX expression between the 71 PBM cases and 19 control cases (Fig. 3A). The median positive proportions of γ-H2AX were 14.4 % (range: 0.8–70.7 %) and 4.4 % (range: 0.7–50.0 %), respectively, and significantly higher for PBM (*p* = 0.001). To assess the involvement of DSBs in the carcinogenic process of PBM, the positive proportions of γ-H2AX were determined in the 14 PBM cases with gallbladder adenocarcinoma and 57 cases without (Fig. 3B). The median positive proportions of γ-H2AX in cases with and without carcinoma were 21.4 % (range: 8.6–48.9 %) and 11.0 % (range: 0.8–70.7 %), respectively, and significantly higher in cases with carcinoma (*p* = 0.031).

### p53 expression was insignificant in this population

As the central and representative tumor suppressor gene, p53 protects the genome by orchestrating a variety of DDR mechanisms [19]. Erroneous DNA repair induces mutations of p53, leading to a loss of normal tumor suppressor function and ensuing cancer development. The abnormal accumulation of mutant p53 can be detected pathologically by p53 immunostaining. We investigated the expression of p53 in PBM and control cases to evaluate the influence of DDR failure on carcinogenesis. However, no cases in either group exhibited detectable p53 expression.

### γ-H2AX expression was stronger in PBM cases without dilation

**Fig. 4:**
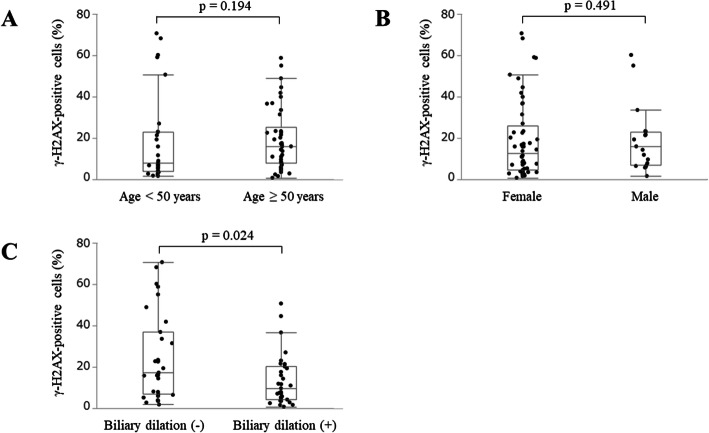
Associations between the degree of γ-H2AX expression and clinical characteristics. There were no significant differences for (**A**) age or (**B**) sex. **C** The expression of γ-H2AX was significantly higher in cases without biliary dilation than in those with dilation (*p* = 0.024). γ-H2AX, phosphorylated H2AX at Ser139

Lastly, we searched for associations between the degree of γ-H2AX expression and clinical characteristics in PBM cases (Fig. 4). No notable differences were observed for age or sex. However, the expression of γ-H2AX was significantly higher in PBM cases without biliary dilation than in those with dilation (*p* = 0.024), with respective median positive proportions of 17.3 % (range: 1.9–70.7 %) and 9.7 % (range: 0.8–50.7 %).

## Discussion

DSBs are the most lethal type of DNA damage and result from a variety of events, including ionizing radiation, ultraviolet light, drugs, and inflammatory attacks [6]. γ-H2AX foci have been reported to be produced very soon after treatment with chemicals. Because this damage is undetectable by the neutral comet assay, γ-H2AX is considered the most sensitive and specific marker of DNA damage caused by various agents [20]. The present study revealed that the expression of γ-H2AX was more prominent in gallbladder epithelium specimens from PBM patients in comparison to controls, indicating that DSBs occurred more frequently in PBM. In PBM, pancreatic juice is constantly refluxed into the biliary duct to produce activated pancreatic enzymes and secondary bile acid. Since these substances are extremely harmful to the biliary epithelium, the tissue is subjected to long-term inflammatory damage [1, 2]. Chronic inflammation can produce active oxygen and nitrogen, which cause cell cycle acceleration, DNA damage, and ultimately DSBs [9, 10]. In a previous study, the immunohistochemical expression of 8-hydroxy-2’-deoxyguanosine, a marker of oxidative damage, confirmed that oxidative injury occurred more prominently in the gallbladder epithelium of subjects with PBM than in those without [21]. In addition, several reports have used immunohistochemical methods to describe the proliferative hyperactivity of the mucosal epithelium in PBM [1, 2, 22, 23]. Ki-67, a common marker of cell proliferation activity, was reportedly overexpressed in the gallbladder epithelium of PBM patients. Thus, the longstanding inflammatory injury in PBM induces various undesirable cell changes, such as oxidative stress and cell proliferation, which elicit repeated DSBs and other serious DNA damage.

In our study, the expression of γ-H2AX was significantly more prominent in the background epithelium of carcinoma in PBM patients in comparison to non-carcinoma cases, indicating that DSBs exist more abundantly in the PBM mucosa surrounding cancer. The background mucosa of cancers is suspected to be more susceptible to oncogenic transformation. The present study showed a close association between DSB formation and PBM peri-cancerous regions, suggesting that a DSB increase could contribute to carcinogenesis in PBM. PBM patients have a reported 200-fold increased risk of biliary duct cancer in comparison to the general population [24]. In a nationwide Japanese survey of 2561 adult PBM patients [24], biliary tract cancers were found in 21.6 % of patients with biliary dilation and in 42.4 % of those without. Specifically, bile duct and gallbladder carcinomas were respectively detected in 6.9 and 13.4 % of PBM patients with biliary dilation and in 3.1 and 37.4 % of those without. The incidence of gallbladder cancer was notably high in PBM patients. Genomic instability is a common characteristic of most cancers and plays a critical role in carcinogenesis [25]. DSBs have been related to the early stage of carcinogenesis in various cancers, and tissues exposed to DSBs are suspected as precancerous lesions [13]. Chronic inflammation of the gallbladder epithelium caused by refluxed pancreatic juice induces DNA damage in PBM patients. In the present study, a large amount of DSBs were specifically detected in the gallbladder mucosa surrounding cancers, implying that DSBs may be related to the early process of gallbladder cancer development. In addition, the histological images of the cancerous portion of PBM, which had stronger expression compared with the non-cancerous portion, may support this hypothesis.

This investigation immunohistochemically evaluated the expression of p53 as a representative tumor suppressor mechanism in the PBM mucosa. However, no cases exhibited detectable p53 expression. A human cell line experiment [26] found that most γ-H2AX foci disappeared within 24 h after irradiation, indicating that the DDR is immediately activated for DNA repair of DSBs in the nucleus. Indeed, the DDR is an essential protective mechanism against malignant cell transformation [5, 7], and its failure to repair DSBs leads to cancer development. Several studies have found that while p53 overexpression was frequently noted in the PBM gallbladder cancerous epithelium, it was largely negative noncancerous lesions, in agreement with our findings [23, 27]. The mutation of p53 is widely recognized as a late event in the adenoma-carcinoma sequence of colorectal cancer. Similarly, in gallbladder cancer with PBM, p53 mutation may be a relatively late event in the carcinogenic process. This study indicated that while DSBs may trigger cancer development as an early event in PBM carcinogenesis, DSBs alone could not initiate oncogenesis. The underlying mechanism of cancer development after DSBs remains unclear, and further study is required to discern the processes that precipitate DDR dysfunction.

PBM is well known to occur in both dilated or non-dilated extrahepatic bile duct forms [1]. The site of cancer development in these two types is quite different. PBM patients without biliary dilation have a high incidence of gallbladder carcinoma, whereas those with biliary dilation display a high incidence of both gallbladder and bile duct carcinomas. The prevalence of gallbladder adenocarcinoma is considerably higher in PBM patients without biliary dilation [24]. According to our data, DSBs occurred significantly more frequently in the PBM gallbladder mucosa of patients without biliary dilation. Considering the higher prevalence of gallbladder cancer in non-dilated PBM, our finding that DSB occurs more commonly in cases without biliary dilation is convincing. In PBM with biliary dilation, the refluxed pancreatic juice is likely to stagnate in the dilated bile duct and gallbladder. However, in PBM without biliary dilation, the stasis is likely to occur selectively in the gallbladder. This is where long-term inflammatory damage to the mucosa may induce the accumulation of DSBs, resulting in a high prevalence of gallbladder cancer.

This study suggests that DSBs induced by persistent inflammation may be involved in the development of gallbladder cancers in PBM at an early stage of carcinogenesis. It has been reported that tissue samples of ulcerative colitis [28] and chronic hepatitis [29], both widely known as chronic inflammatory diseases predisposing patients to cancer, overexpressed γ-H2AX. γ-H2AX was found to be a marker for predicting carcinogenesis in those diseases. Furthermore, γ-H2AX has been reported as a prognostic factor in several other types of cancers, including breast cancer [30], hepatocellular carcinoma [29], lung cancer [31], and colorectal cancer [32], in which γ-H2AX overexpression has been associated with poor patient survival. Taking the above into consideration, γ-H2AX may have a role in early cancer screening as a prognostic indicator in PBM patients. Larger studies are required to elucidate the detailed role of this marker. Because there are some reports that have used immunohistochemistry [18], we chose to evaluate γ-H2AX expression using this technique. However, analysis by immunohistochemistry is not common, and analysis of γ-H2AX expression by immunofluorescence may find new facts. Therefore, future analysis with immunofluorescence is warranted.

## Conclusions

DSBs occurred more abundantly in the PBM gallbladder mucosa, especially in the background mucosa of cancers, which implicates DSB involvement in early PBM carcinogenesis. The diagnostic and prognostic utility of γ-H2AX for tumors in patients with PBM merits greater attention.

## Data Availability

All data generated and analyzed during the current study are available from the corresponding author on reasonable request.

## Abbreviations

PBM: Pancreaticobiliary maljunction
DSBs: DNA double-strand breaks
DDR: DNA damage response
γ-H2AX: Ser139 phosphorylation of histone H2AX

## References

[1] Kamisawa T, Kuruma S, Chiba K, Tabata T, Koizumi S, Kikuyama M (2017). Biliary carcinogenesis in pancreaticobiliary maljunction. J Gastroenterol.

[2] Tsuchida A, Itoi T (2010). Carcinogenesis and chemoprevention of biliary tract cancer in pancreaticobiliary maljunction. World J Gastrointest Oncol.

[3] Jeong IH, Jung YS, Kim H, Kim BW, Kim JW, Hong J (2005). Amylase level in extrahepatic bile duct in adult patients with choledochal cyst plus anomalous pancreatico-biliary ductal union. World J Gastroenterol.

[4] Kamisawa T, Ando H, Suyama M, Shimada M, Morine Y, Shimada H (2012). Japanese clinical practice guidelines for pancreaticobiliary maljunction. J Gastroenterol.

[5] Bartkova J, Horejsi Z, Koed K, Kramer A, Tort F, Zieger K (2005). DNA damage response as a candidate anti-cancer barrier in early human tumorigenesis. Nature.

[6] Bonner WM, Redon CE, Dickey JS, Nakamura AJ, Sedelnikova OA, Solier S (2008). GammaH2AX and cancer. Nat Rev Cancer.

[7] Gorgoulis VG, Vassiliou LV, Karakaidos P, Zacharatos P, Kotsinas A, Liloglou T (2005). Activation of the DNA damage checkpoint and genomic instability in human precancerous lesions. Nature.

[8] McKinnon PJ, Caldecott KW (2007). DNA strand break repair and human genetic disease. Annu Rev Genomics Hum Genet.

[9] Murata M, Thanan R, Ma N, Kawanishi S (2012). Role of nitrative and oxidative DNA damage in inflammation-related carcinogenesis. J Biomed Biotechnol.

[10] Ohnishi S, Ma N, Thanan R, Pinlaor S, Hammam O, Murata M (2013). DNA damage in inflammation-related carcinogenesis and cancer stem cells. Oxid Med Cell Longev.

[11] Balkwill F, Charles KA, Mantovani A (2005). Smoldering and polarized inflammation in the initiation and promotion of malignant disease. Cancer Cell.

[12] Coussens LM, Werb Z (2002). Inflammation and cancer. Nature.

[13] Jeggo PA, Lobrich M (2007). DNA double-strand breaks: their cellular and clinical impact?. Oncogene.

[14] Rogakou EP, Pilch DR, Orr AH, Ivanova VS, Bonner WM (1998). DNA double-stranded breaks induce histone H2AX phosphorylation on serine 139. J Biol Chem.

[15] Bassing CH, Chua KF, Sekiguchi J, Suh H, Whitlow SR, Fleming JC (2002). Increased ionizing radiation sensitivity and genomic instability in the absence of histone H2AX. Proc Natl Acad Sci.

[16] Sedelnikova OA, Rogakou EP, Panyutin IG, Bonner WM (2002). Quantitative detection of (125)IdU-induced DNA double-strand breaks with gamma-H2AX antibody. Radiat Res.

[17] Kamisawa T, Ando H, Hamada Y, Fujii H, Koshinaga T, Urushihara N (2014). Diagnostic criteria for pancreaticobiliary maljunction 2013. J Hepatobiliary Pancreat Sci.

[18] Sato Y, Kubo S, Takemura S, Sugawara Y, Tanaka S, Fujikawa M (2014). Different carcinogenic process in cholangiocarcinoma cases epidemically developing among workers of a printing company in Japan. Int J Clin Exp Pathol.

[19] Williams AB, Schumacher B. p53 in the DNA-Damage-Repair Process. Cold Spring Harb Perspect Med 2016;6. 10.1101/cshperspect.a026070 PMC4852800 27048304

[20] Yu Y, Zhu W, Diao H, Zhou C, Chen FF, Yang J (2006). A comparative study of using comet assay and gammaH2AX foci formation in the detection of N-methyl-N’-nitro-N-nitrosoguanidine-induced DNA damage. Toxicol In Vitro.

[21] Otani K, Shimizu S, Chijiiwa K, Yamaguchi K, Noshiro H, Tanaka M (2001). Immunohistochemical detection of 8-hydroxy-2’-deoxyguanosine in gallbladder epithelium of patients with pancreaticobiliary maljunction. Eur J Gastroenterol Hepatol.

[22] Seki M, Yanagisawa A, Ninomiya E, Ninomiya Y, Ohta H, Saiura A (2005). Clinicopathology of pancreaticobiliary maljunction: relationship between alterations in background biliary epithelium and neoplastic development. J Hepato-Bilia-Pancr Surg.

[23] Ichikawa Y, Kamiyama M, Sekido H, Ishikawa T, Miura Y, Kamiya N (2004). Telomerase activity and Bcl-2 expression in gallbladders of pancreaticobiliary maljunction patients: a preliminary study. J Hepato-Bilia-Pancr Surg.

[24] Morine Y, Shimada M, Takamatsu H, Araida T, Endo I, Kubota M (2013). Clinical features of pancreaticobiliary maljunction: update analysis of 2nd Japan-nationwide survey. J Hepatobiliary Pancreat Sci.

[25] Stratton MR, Campbell PJ, Futreal PA (2009). The cancer genome. Nature.

[26] Markova E, Schultz N, Belyaev IY (2007). Kinetics and dose-response of residual 53BP1/gamma-H2AX foci: co-localization, relationship with DSB repair and clonogenic survival. Int J Radiat Biol.

[27] Nagai M, Watanabe M, Iwase T, Yamao K, Isaji S (2002). Clinical and genetic analysis of noncancerous and cancerous biliary epithelium in patients with pancreaticobiliary maljunction. World J Surg.

[28] Risques RA, Lai LA, Brentnall TA, Li L, Feng Z, Gallaher J (2008). Ulcerative colitis is a disease of accelerated colon aging: evidence from telomere attrition and DNA damage. Gastroenterology.

[29] Matsuda Y, Wakai T, Kubota M, Osawa M, Takamura M, Yamagiwa S (2013). DNA damage sensor gamma -H2AX is increased in preneoplastic lesions of hepatocellular carcinoma. Sci World J.

[30] Nagelkerke A, van Kuijk SJ, Sweep FC, Nagtegaal ID, Hoogerbrugge N, Martens JW (2011). Constitutive expression of gamma-H2AX has prognostic relevance in triple negative breast cancer. Radiother Oncol.

[31] Matthaios D, Foukas PG, Kefala M, Hountis P, Trypsianis G, Panayiotides IG (2012). gamma-H2AX expression detected by immunohistochemistry correlates with prognosis in early operable non-small cell lung cancer. Onco Targets Ther.

[32] Lee YC, Yin TC, Chen YT, Chai CY, Wang JY, Liu MC (2015). High expression of phospho-H2AX predicts a poor prognosis in colorectal cancer. Anticancer Res.

